# Discovering Anti-Cancer Drugs *via* Computational Methods

**DOI:** 10.3389/fphar.2020.00733

**Published:** 2020-05-20

**Authors:** Wenqiang Cui, Adnane Aouidate, Shouguo Wang, Qiuliyang Yu, Yanhua Li, Shuguang Yuan

**Affiliations:** ^1^Shenzhen Institutes of Advanced Technology, Chinese Academy of Sciences, Shenzhen, China; ^2^College of Veterinary Medicine, Northeast Agricultural University, Harbin, China

**Keywords:** anti-cancer, CADD, drug discovery, AI, computational methods

## Abstract

New drug discovery has been acknowledged as a complicated, expensive, time-consuming, and challenging project. It has been estimated that around 12 years and 2.7 billion USD, on average, are demanded for a new drug discovery *via* traditional drug development pipeline. How to reduce the research cost and speed up the development process of new drug discovery has become a challenging, urgent question for the pharmaceutical industry. Computer-aided drug discovery (CADD) has emerged as a powerful, and promising technology for faster, cheaper, and more effective drug design. Recently, the rapid growth of computational tools for drug discovery, including anticancer therapies, has exhibited a significant and outstanding impact on anticancer drug design, and has also provided fruitful insights into the area of cancer therapy. In this work, we discussed the different subareas of the computer-aided drug discovery process with a focus on anticancer drugs.

## Introduction

Up to now, cancer remains a global and serious public health challenge. It is estimated that there are more than 200 different types of cancer, generally named according to the tissue where the cancer was recognized for the first time. Cancer is considered to be one of the significant causes for death in the 21st century and the most critical obstacle for the increase of global life expectancy. According to an analysis by the world health organization (WHO) in 2015, cancer is the second leading cause of death for patients younger than 70 years old in 91 countries and the third or fourth leading cause of death among 22 other countries (Yan et al., 2019). Moreover, a global increase of 18.1 million new cancer cases and 9.6 million cancer-related deaths have been reported in a previous study (Bray et al., 2018), especially 70% of the death caused by cancer occur in low-income and middle-income countries. The fast growth of the cancer incidence and mortality has turned out to be global health challenges. How to reduce the cancer-related death rate has attracted significant attention from the government, society, medical industry, as well as scientific communities, expecting the rapid development of effective and safe drugs for cancer treatment.

Despite of the impressive progress in biotechnologies and further understandings of the disease biology, the development of new, practical and innovative small molecule drugs remains an arduous, time-consuming, and expensive project, which requires collaborations from many expertise in multidisciplinary fields, including medicinal chemistry, computational chemistry, biology, drug metabolism, clinical research, etc. Furthermore, it has been illustrated that the successful discovery and development of a new drug costs 12 years, and expensive investment (Kapetanovic, 2008). Thus, novel drug development strategies with a reduced cost of time and money, as well as an enhanced efficiency are in high demand, which would contribute to a significant improvement in global health and life expectancy. Since the successful development of HIV protease inhibitor Viracept in the USA in 1997, which was the first drug design fully driven by its target structure (Kaldor et al., 1997), computational methods have served as an essential tool in drug discovery projects and have been a cornerstone for new drug development approaches. This makes the drug developmental process faster and cheaper. Recently, the fast growth in computational power, including massively parallel computing on graphical processing units (GPUs), the continuous advances in artificial intelligence (AI) tools (Chan et al., 2019; Yang et al., 2019), have translated fundamental research into practical applications (Zhavoronkov et al., 2019) in the drug discovery field. This attracted considerable attention for their outstanding performance on providing new promising perspectives and solutions to overcome life-threatening diseases.

**Figure 1:**
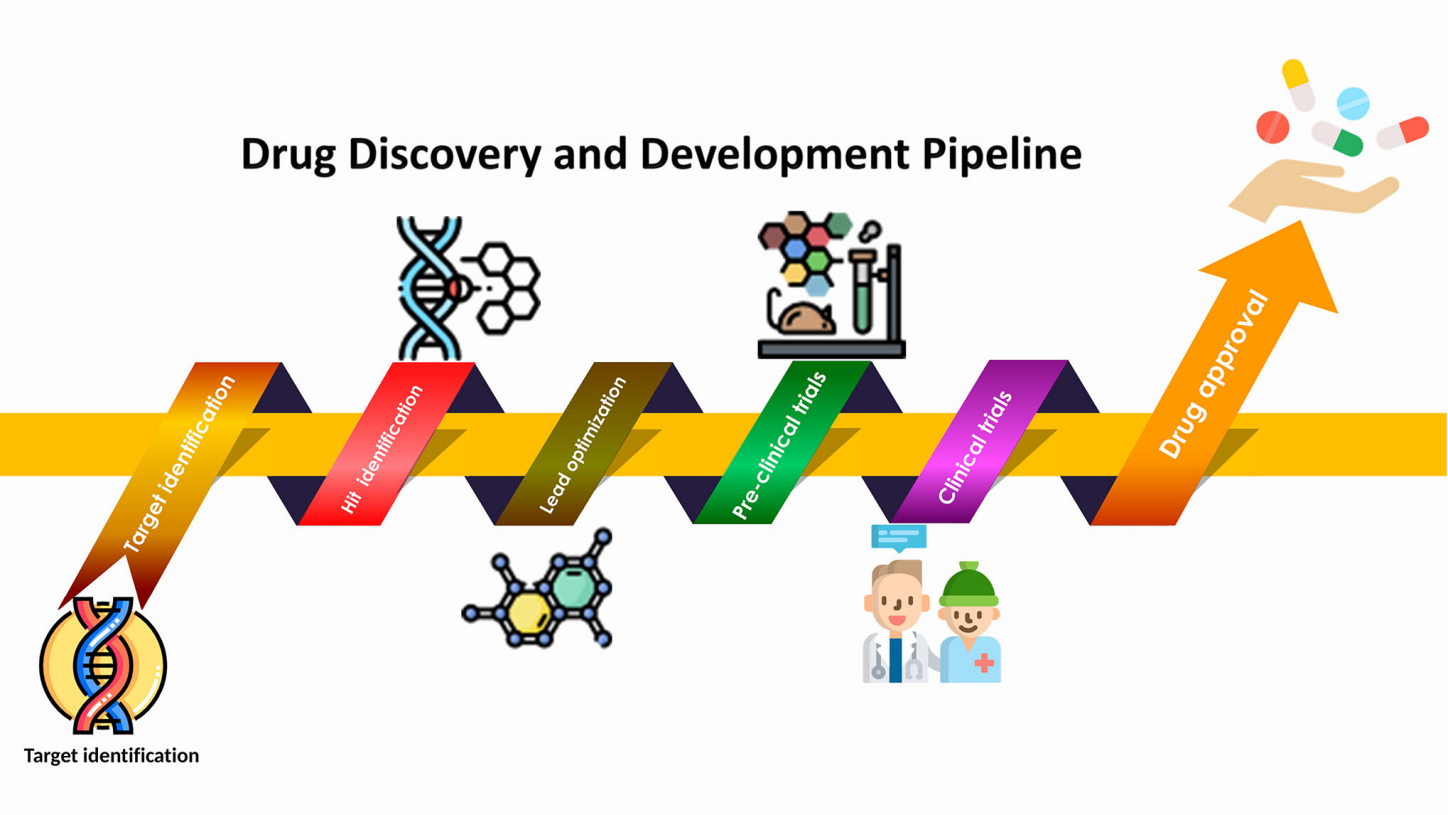
A workflow for drug discovery: from target identification to drug approval.

In this review, we aim at providing an overview of different subjects of the computational-method-aided new drug discovery processes in general, and anti-cancer therapy discovery in particular. We reviewed some of the most representative examples and clarified fundamental principles by exploring studies on anticancer drug designs with the help of computational methods. A workflow of computational drug discovery is explained in **Figure 1**.

## Anti-Cancer Drug Target Prediction

Human contains approximately 30,000 genes, among which around 6,000 to 8,000 sites are estimated as potential pharmacological targets. However, less than 400 encoded proteins have been proved to be effective for drug development until now (Drews, 2000; Chen et al., 2016). Cancer, compared to many other human diseases, now has a plethora of potential molecular targets for therapeutic development (Lazo and Sharlow, 2016). Traditional drug discovery mainly follows the paradigm of “one molecule - one target - one disease”, without considering the interactions between drugs and proteins. However, an important fact that many complex diseases are relevant to a variety of target proteins (Hopkins, 2008; Yamanishi et al., 2008; Chen et al., 2012) has been overlooked. Furthermore, unexpected drug functions derived from off-targets are an accidental and uncontrollable activities because of the “poly-pharmacological” properties of certain drugs, which might result in undesirable side effects. Those are particularly pronounced for cancer drugs. On the other hand, there are some positive examples that benefit from the different pathways targeted by one given molecule. For example, sildenafil (viagra) was developed to treat angina, but now it is used for erectile dysfunction therapy (Ghofrani et al., 2006). There are several drugs, including anticancer drugs, whose corresponding target proteins (both primary and non-target) remain yet unidentified or unknown (Takarabe et al., 2012). Furthermore, some attractive and potentially effective cancer targets remain outside of the scope of pharmacological regulation. Some of these targets such as phosphatases, transcription factors, and RAS family members have been described as undruggable, as they lack effective enzymatic active sites (Lazo and Sharlow, 2016). To make the full use of known drugs to treat new indications, the characterization of all potential new ligand binding sites has been illustrated as a key point in drug repositioning and repurposing. Therefore, new and highly qualitative bioinformatic target prediction methods are required for the accurate prediction of drug targets.

Up to now, a wide range of drug target interactive web servers has been established, providing a series of drug-target databases and prediction tools (see **Tables 1** and **2**). Moreover, various computational approaches have been used to study potential interactions between proteins and drugs. In particular, network-based models and ML-based models have emerged as important tools. A review by Chen et al. summarizes several available computational models for this application (Chen et al., 2016). Interestingly, a method proposed by Campillos et al. that uses the similarity of drug side effects to determine whether multiple drugs could interact with the same target proteins attracted our attention (Campillos et al., 2008). Based on this research, Takarabe et al. took advantage of the US FDA's adverse event reporting system (AERS) to define the pharmacological similarity of all potential medicines and developed a novel system to predict large-scale interactions between unknown drug-targets (Takarabe et al., 2012). Notably, AERS was employed to predict interactions between drugs and targets for the first time. In 2010, Klipp et al. summarized several available computational models for network-based drug-target prediction (Klipp et al., 2010). Moreover, various biological data settings, including structures of bioactive compounds, sequences of target proteins, and information of ligand-target interactions, have been combined. A series of machine learning-based approaches have been demonstrated as efficient tools in detecting relationships among drug structures and corresponding target proteins from a large amount of data, such as supervised learning method (Srivastava et al., 2014), bipartite graph learning method (Li and Chen, 2013), bipartite local model (Yildirim et al., 2007), and so on. A recent review by Mayr et al. compared the predictive performance of deep learning with other prediction approaches for multiple drug targets in the comparative studies of composite target prediction methods. As a result, feed-forward neural networks were identified with better performance in drug target prediction than other methods (Mayr et al., 2018).

**Table 1:** Drug-target database.

Databases	Websites
DrugBank	https://www.drugbank.ca/
TTD	http://bidd.nus.edu.sg/group/ttd/ttd.asp
MATADOR	http://matador.embl.de/
SuperTarget	http://insilico.charite.de/supertarget/
TDR targets	http://tdrtargets.org/
PDTD	http://www.dddc.ac.cn/pdtd/
ChEMBL	https://www.ebi.ac.uk/chembldb
STITCH	http://stitch.embl.de/
BindingDB	http://www.bindingdb.org/
CancerDR	http://crdd.osdd.net/raghava/cancerdr/
DCDB	http://www.cls.zju.edu.cn/dcdb/

**Table 2:** Computational tools for target prediction.

Computational tools	Websites
SEA	https://omictools.com/sea-2-tool
Pharmmapper	http://www.lilab-ecust.cn/pharmmapper/
Chemmapper	https://omictools.com/chemmapper-tool
Tide	http://sysbio.molgen.mpg.de/tide
DINIES	http://www.genome.jp/tools/dinies/
SuperPred	http://prediction.charite.de/
SwissTarget Prediction	http://www.swisstargetprediction.ch/

As above, since a large number of compounds and vigorous efforts are abandoned and wasted due to the off-target effects during the classical drug development procedure, a greatly enhanced development of target prediction in new drug exploration exhibited attractive advantages and further expansion in this area are still highly desirable

## Structure-Based Drug Discovery

Structure-based strategy relies on the known structural information to define the interaction effect between bioactive compounds and the corresponding receptors. (Wang et al., 2000). With the development of biomolecular spectroscopic technologies such as X-ray crystallography and nuclear magnetic resonance (NMR), remarkable progress has been made in this field, leading to considerable improvements in our structural understanding of the drug target. Taking advantages of the three-dimensional structure of the proteins, new ligands could be rationally designed to trigger therapeutic effects. Hence, structure-based design (SBD) could provide critical insights into new drug design and development *via* discovering and optimizing the initial lead compounds (Prada-Gracia et al., 2016; Lu et al., 2018a). The high affinity ligand regulates validated drug targets selectively to influence specific cellular activities, ultimately achieving the desired pharmacological, and therapeutic effects (Urwyler, 2011). Capoten (captopril), the first ACE (angiotensin-converting enzyme) inhibitor, was one of the first successful examples of using structural information to optimize drug designs in the 1980s (Anthony et al., 2012). Since this study, structure-based drug development started to serve as a novel and powerful algorithm and technique to promote faster, cheaper, and more effective drug development. In the past decade, extensive efforts have been made to promote the strategy of SBD, more and more successful applications played important roles in new medical research (Debnath et al., 2019; Hong et al., 2019; Mendoza et al., 2019; Itoh, 2020; Tondo et al., 2020).

### Molecular Docking

Molecular docking is a typical structure-based protocol in rational drug design by studying and predicting the binding patterns and interaction affinities among the ligand and receptor biomolecules (Ferreira et al., 2015). It could be categorized as rigid docking and flexible docking according to the flexibility of the ligands involved in the computational process (Halperin et al., 2002; Dias and De Azevedo, 2008). The rigid docking method is a binding model which only considers the static geometrical, physical, and chemical complementarity between the ligand and the target proteins, while ignores the flexibility and the induced-fit theory (Salmaso and Moro, 2018). In general, the rigid docking, which is fast and highly effective, is applied to the high throughput virtual screening with a large number of small-molecule databases to be time-efficient. While the flexible docking method considers more detailed and accurate information. With the rapid improvement of computing resources and efficiency, flexible docking methods developed continuously and became more easily accessible. There are different types of software available for docking, such as Glide, FlexX, DOCK, AutoDock, Discovery Studio, Sybyl, etc.

The molecular docking process is mainly composed of three steps. First, the structures of small molecules and target proteins should be prepared in advance. In this step, abundant experimentally solved structures are available in the open access PDB database (http://www.rcsb.org), which can be used to understand many physiological processes based on the crystal structures, and also for homologous template models if docking structures are of interest. Second, it can act as an engine for predicting conformations, orientations, and positional spaces in the ligand binding site (Mathi et al., 2018). Conformational search algorithms carry out this task to predict the conformations of binary complexes by applying the methods of systematic and stochastic search. Systematic search techniques include: (i) Exhaustive search; (ii) Fragmentation; (iii) Conformational Ensemble. On the other hand, stochastic methods include: (i) Monte Carlo (MC) methods; (ii) Tabu search methods; (iii) Evolutionary Algorithms (EA); (IV) Swarm optimization (SO) methods (Ferreira et al., 2015). Finally, these programs evaluate the putative binding-free energy, which associates the scoring function to determine which compounds are more likely to bind to targets during the molecular docking (Huang et al., 2010). There are four essential types of scoring functions, including: (i) Consensus scoring functions (ii) Empirical scoring functions; (iii) Knowledge-based scoring functions; (iv) Force-field based scoring functions (Kortagere and Ekins, 2010). Furthermore, new scoring capabilities have been developed, for example (i) machine learning technologies; (ii) interactive fingerprints; (iii) quantum mechanical scores (Yuriev et al., 2015).

### Structure-Based Pharmacophore Mapping

With the development in the past decades, the pharmacophore mapping method has been considered as one of the most useful technology during the process of drug discovery. All kinds of structure-based approaches have been conducted to improve pharmacophore modeling, which has been widely used for virtual screening*, de novo* design as well as lead optimization (Yang, 2010; Lu et al., 2018a). The structure-based pharmacophore (SBP) is another useful method. Based on the availability of ligand structures, SBP modeling methods can be cataloged into two types: target-ligand complex-based methods and target-binding site-based (without ligand) methods (Pirhadi et al., 2013). The approach based on the target-ligand complex can conveniently locate the ligand-binding pocket of the protein and assess the main ligand-protein interactions. This is exampled by LigandScout (Wolber et al., 2006), Pocket v.2 (Chen and Lai, 2006), and GBPM (Ortuso et al., 2006). It is worth noting that they cannot be used to the situations where ligands are unknown. The macromolecule (without ligand)-based method implemented in Discovery Studio (Lu et al., 2018b) is an obvious example which is not dependent on the ligands and the receptor-ligand interactions. The LUDI program (Bohm, 1992) defines the interactions within the binding site as pharmacological characteristics. Although this purely SBP method has the advantage of describing the entire interaction capability of a binding pocket, the main limitation of this method is that the derived interaction maps typically involve many unprioritized interaction features.

## Ligand-Based Drug Discovery

### Similarity Searching

The main principle and motivation behind the ligand-based approaches in drug discovery is a concept known as molecular similarity; based on this principle, molecules tend to perform similar biological effects due to the high structural similarity (Zhavoronkov et al., 2019). In other words, ligand-based drug discovery methods rely on the structural information of the active ligand that interacts with the target protein, and such a compound with interesting biological properties can be used as a query template in identifying and predicting new chemical entities with similar properties. Since only the structure of the known active small molecules are required, this methodology is considered as an indirective protocol for drug discovery. It offers an option when the 3D target protein structure is unknown or cannot be predicted. Hence, this approach is commonly applied to screen novel ligands with interesting biological activities *in silico* and to optimize the biological activities of ligands to improve drug pharmacokinetics including Adsorption, Distribution, Metabolism, Excretion, Toxicity (ADMET) properties.

This simple and most widely used technique is based on molecular descriptors. Physicochemical properties (e.g., molecular weight, logP, Energy of high occupied molecular orbital (EHOMO), Energy of lowest unoccupied orbital (ELUMO), charges), as well as 2D fingerprint and 3D shape-similarity searches can be introduced as coordinates to represent the reference compounds. The 2D fingerprint (Molprint2D and Unity 2D) and 3D shape similarity methods (MACCS), extended-connectivity fingerprints (ECFP), rapid overlay of chemical structures (ROCS), and Phase Shape, are more often used for molecular representation in virtual screening (Rush et al., 2005). For example, Bologa et al. (2006) applied 2D fingerprint and 3D shape-similarity methods to identify novel agonists of the estradiol receptor family receptor GPR30 (Bologa et al.). Furthermore, both methods have been successfully applied in virtual screenings, and both technology have exhibited better performance against a number of targets than docking methods in terms of the scalability and computational time. However, the main problem of the similar methods is their preference for input molecules and the difficulty in deciding which input structures to be used (Hu et al., 2012).

### Ligand-Based Pharmacophore Mapping

Another more precise approach in comparison with the molecular descriptors is the pharmacophore-based approach, in which a pharmacophore model (PH4) is developed based on a group of active compounds. The IUPAC (International Union of Pure and Applied Chemistry) pointed out that a pharmacophore is “a collection of spatial and electronic characteristics necessary to ensure optimal supramolecular interactions with specific biological targets and to trigger (or block) their biological reactions” (Buckle et al., 2013). Thus, structural overlap of key molecular features derived from active compounds or a binding site in space are used as a pattern to represent the most probable chemical characteristics. The newly identified molecules that match and show a high complementation to the developed pharmacophore are likely to be active against the target protein of interests. Therefore, they can be selected as candidates for more further investigations. This approach has become a key computational strategy to promote and guide drug discoveries in the absence of macromolecular structures (Chao et al., 2007).

The process of pharmacophore modelling can be summarized as following: (i) Selection of a training set of ligands (active and inactive compounds). (ii) Molecular preparation (low energy conformations). (iii) Ligand alignment/superimposition and pharmacophore model generation. (iv) Validation of pharmacophore models (Chiang et al., 2009). Ligand-based pharmacophore modeling highly depends on the availability of a good training set of compounds manifesting the same binding mode.

### QSAR Modeling

QSAR (Quantitative Structure Activity Relationship) is another ligand-based approach that relies on analyzing the biological activities of drugs using various molecular descriptors (MDs) or fingerprints (FPs). These models mathematically describe how the activities response to the targets according to the ligand's structural characteristics. QSAR was obtained by calculating the correlations between the properties of the ligand binding agent and the biological activity measured by experiments. Different ML and deep learning (DL) approaches have also been applied to develop QSAR models (Mendenhall and Meiler, 2016): including Support Vector Machine (SVM), Random Forest (RF), Polynomial Regression (PR), Multi Linear Regression (MLR), Artificial Neural Network (ANN). Unlike the pharmacophore models, QSAR models can measure biological activities quantitatively and can even find positive or negative effects according to certain characteristics of the molecule on its activity.

QSAR has been applied to many other molecular design purposes, such as predicting the new molecule analog activity, optimizing lead, and predicting new structural leads in drug discovery. In the classical 2D-QSAR approaches, the biological activity is related to physical and chemical features consisting of steric, electronic, and hydrophobic characters of drugs, and the relationships are represented as mathematical equations (Hansch and Fujita, 1964). More advanced 3D-QSAR approaches, such as comparative molecular field analysis (Cramer et al., 1988) and molecular similarity indexes in a comparative analysis (Klebe et al., 1994), are based on the force field calculations. The structural information of molecules is needed, and developed models are represented in 3D contour maps facilitating the visualization and interpretation.

## Using MD simulation to Find New Drug Binding Sites

Many important biological events rely on the information of protein-ligand complex interactions. The recognition and characterization of LBP is the key to understand the function of endogenous ligands and synthetic drug molecules. GPCRs perform an important role in a variety of physiological processes. GPCRs are a class of commonly used targets in drug discovery (Conn et al., 2009). Recent discovery indicated that beside binding to orthosteric sites, ligands could bind to different allosteric sites that are far away from the targeted binding pockets (Tautermann, 2014; Flock et al., 2015; Devree et al., 2016). Unfortunately, the position of such allosteric pocket is unclear without the information of experimental structures, and predicting the existence of such sites could facilitate the discovery of new drugs (Tautermann, 2014). A recent overview described the progresses in important computational tools for the prediction of functional sites, such as 3DLigandStie (http://www.sbg.bio.ic.ac.uk/~3dligandsite/), COACH-D (http://yanglab.nankai.edu.cn/COACH-D/), or SiteMap (https://www.schrodinger.com/sitemap), and many others. However, these reported tools often create multiple possible ligand binding sites, and sometimes it is not easy for the user to confirm which active pocket is real one for the compound binding. To overcome this limitation, methods based on molecular dynamics (MD) have been developed in recent years. For example, the supervised MD is an efficient approach for precise sampling and the identification of ligand-binding sites (Sabbadin and Moro, 2014; Deganutti et al., 2015; Cuzzolin et al., 2016). The conventional long-timescale MD has also been successfully applied for new drug binding sites (Chan et al., 2018). Similarly, a study by Chan et al. (2020) reported that an additional sodium ion, which located in the vicinity of the orthosteric binding site, by MD simulations (Chan et al., 2020). MD could also be applied for the recognition of the allosteric sites involved in protein kinases (Tong and Seeliger, 2015), Ras proteins (Hancock, 2003), and *Staphylococcus aureus* Sortase A (Mazmanian et al., 1999). As above, information obtained from MD predictions provides new opportunities of drug discovery.

## Artificial Intelligence in Anti-Cancer Drug Discovery

Computational drug design has successfully promoted the discovery of several new anticancer drugs, which has become a milestone in this area. Gefitinib (Muhsin et al., 2003), Erlotinib (Grunwald and Hidalgo, 2003), Sorafenib (Wilhelm et al., 2006), Lapatinib (Wood et al., 2004), Abiraterone (Jarman et al., 1998), Crizotinib (Butrynski et al., 2010) are all approved drugs that have been discovered based on computational drug methods. Until now, the anticancer drug research is rapidly progressing: computational, and AI methods are generating new promising results. As an example, SR13668 is optimized from indole-3-carbinol (I3C) using PH4 design. SR13668 has shown a strong effect on different cancers in phase I (Chao et al., 2007). Recently, Rodrigues et al. have successfully identified a potent inhibitor for 5-lipoxygenase by using machine learning (ML)-based method which was developed from physicochemical and pharmacophore characteristics (Reker et al., 2014; Rodrigues et al., 2018). With the arrival of AI, the design of anticancer drugs *in silico* has undergone unprecedented changes, and state-of-the-art deep learning approaches have the potential to produce the excellent chemical properties needed for new molecules (Gomez-Bombarelli et al., 2018). Similarly, Jann et al. have developed the first ML-based anti-cancer compound generator using variational autoencoders (VAEs) and have demonstrated that the compound production may be selective toward molecules with high predicted inhibition to a specific cancer (Born et al., 2019). This implied that models could be developed to yield drug candidates with highly desired efficacy (IC_50_) against a target of interest. This breakthrough could transform the design of anticancer drugs *in silico* by taking advantage of the bimolecular features of the disease to improve the success rate of lead compound discovery.

## Successful Stories of Computational Drug Discovery

Computational methods have proved to play an essential role in modern drug discovery. Since computational methods could cover almost all stages of the drug discovery pipeline, the applications of computational methods in anticancer drug discoveries have shown great advantages in terms of the required investment, resources, and time. More recently, computational methods have become a potent and powerful tool in several successful cases of anticancer drug development. Herein, we list several successful applications of computational methods for small molecule drugs, which have been applied to cancer treatment or are at later stages in the clinical trial.

The development of Crizotinib is a successful example of applying structure-based design techniques (Cui et al., 2011; Kung et al., 2015). Crizotinib has been considered as a selective and potent cMet/ALK dual inhibitor, which was approved by FDA in 2011 (Cui et al., 2013). c-Met, also known as HGFR (hepatocyte growth factor receptor), and its corresponding natural ligand HGF (hepatocyte growth factor) play a critical role in different cell activities (Christensen et al., 2005). The over-expression of c-Met protein has been often detected in human cancers (including SCLC and NSCLC) (Bottaro et al., 1991; Liu et al., 2008), and abnormal function of c-Met signaling was observed in various solid and blood tumor cancers. Thus, c-MET is an attractive and promising oncology target.

**Scheme 1:**
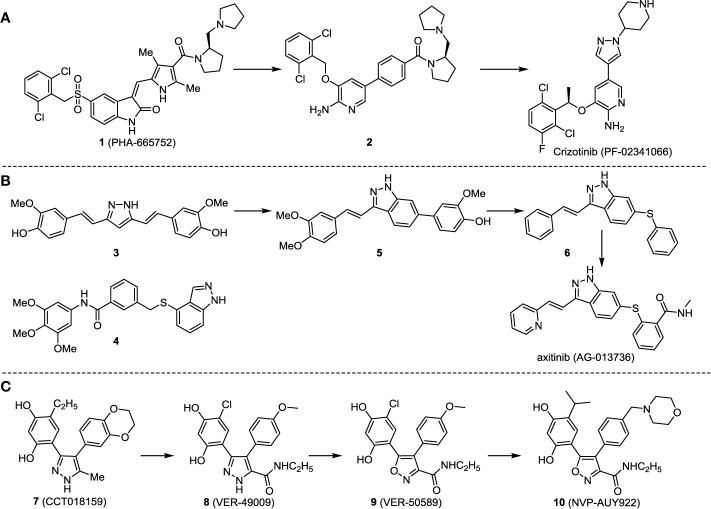
Successful applications of computational methods in anti-cancer drug discovery.

The investigation started with evaluating a series of 3-substituted indolin-2-ones, a potent class of kinase inhibitors, indolin-2-one derivatives for c-MET inhibition. Among the derivatives, compound 1 (PHA-665752, **Scheme 1**) showed strong activity against the c-MET autophosphorylation process and the corresponding biological activations both *in vitro* and *in vivo*. However, the bad drug-like characteristics of compound 1 (PHA-665752) limited its further study. The co-crystal structure analysis of compound 1 with the kinase domain of c-MET elucidated the key inhibitor binding site, presenting opportunities for more efficient drug designs. In combination with re-designing the central rings of compound 1 (PHA-665752), a new set of 5-substituted 3-benzyloxy-2-aminopyridine series has been developed. Among these newly designed derivatives, compound 2 displayed promising inhibition against c-MET. It is noted that lipophilic efficiency (LipE) was employed as the parameter for the binding effectiveness to monitor the progress of optimization. To further improve the c-Met inhibitory potency, a docked structure of compound 2 with the c-Met kinase domain was carried out to guide the application of structure-based design techniques. Followed by optimization of 3-benzyloxy group, the functional group at 5-position of the 2-aminopyridine, and examination of the chiral center, crizotinib (PF-02341066) with effective tumor growth inhibition and good drug performance has been achieved (see **Scheme 1A**). Moreover, Crizotinib has demonstrated remarkable clinical efficacy on c-MET gene amplification against lung cancer, lymphoma, and esophageal cancers (Cui et al., 2011; Lennerz et al., 2011; Schwab et al., 2014).

In 2012, Axitinib (AG-013736) was approved by the FDA as as a new therapy for advanced renal cell carcinoma (Meadows and Hurwitz, 2012) to treat VEHG. Axitinib was developed with a structure-based drug design strategy and served as an inhibitor by binding to the VEGF kinase domain in the DFG-out conformation (Kania, 2009; Kania et al., 2016). The VEGF (vascular endothelial growth factor) family functions as important regulators of many signaling networks which involves in angiogenesis. VEGF signaling was identified in tumor cells, and the VEGF signaling plays a crucial role in the development of malignant diseases. As the key receptors of VEGF, VEGFRs serve as ligands in the VEGF signaling network. The VEGF receptors are known as a class of the tyrosine kinases (RTKs), including VEGFR-1 (also called FLT1), VEGFR-2 (also called FLK1 and KDR) and VEGFR-3 (also called FLT4). Blocking the action of VEGFRs with a pan kinase inhibitor against VEGFR-1, VEGFR-2, and VEGFR-3 has been proved to be an efficient way of anti-angiogenic drug development.

During the developmental process, the crystal structure of phosphorylated construct (p-VEGFR2Δ50), the resolved structures of inhibitor–VEGFR2Δ50 (unphosphorylated kinase) complexes, and robust SAR provided important guidance to the rational drug design (Kania, 2009). Combining with the complex structure information, a collection of compounds has been evaluated, generating pyrazoles 3 and benzamide 4 as the starting point for the drug design. Further efforts have been made by the modeling of pyrazole 3 into the ligand-free p-VEGFR2Δ50 structure to modify the conformation of pyrazole 3 further, leading to the generation of indazole compounds as novel kinase inhibitors. Among these derivatives, compound 5 with a styryl functional group at the 3-position of the indazole ring was identified to exhibit potent inhibitory effect (Ki of 0.3 nM), with a high level of LipE and LE. The crystal structure of VEGFR2Δ50 with compound 5 revealed the detailed enzyme-ligand mode, showing the indazole core binding to the “open” DFG-in conformation of VEGFR2Δ50. Superimposing the other two VEGFR2Δ50–inhibitor co-crystal complex structures demonstrated a more precise 3D structure of the key binding sites for the induction of the DFG-out conformation. Inspired by the superposition result, a chimera design protocol was applied for the subsequent design to capture the above described inhibitor interactions, giving access to 6–sulfur linked indazole compound 6 and the corresponding amide analogs. Further studies on the overlay of VEGFR2Δ50 bound co-crystal structures of benzamide 4 and indazole 6 demonstrated that an additional amide group on the orthosteric site of S-phenyl group would help to make the two important hydrogen bonds with the hydrogen bonding groups from Glu885 and Asp1046 of VEGFR2Δ50 and provide highly potent inhibitors. Further applying the truncation strategy generated axitinib (AG-013736) (see **Scheme 1B**), which exhibited a remarkable improvement on cellular potency, desirable physiochemical, and PK properties. Very recently, axitinib (Inlyta^®^), in combination with pembrolizumab (KEYTRUDA^®^), was approved as the first-line anticancer drug against renal cell carcinoma (RCC)(Atkins et al., 2018).

Heat shock protein 90 (HSP90) has direct and essential effects on the correct performance of different proteins with their activation, conformation, stabilization, and localization functions, whose alterations are associated with cancer development. Thus, HSP90 has become a promising target for cancer treatment (Whitesell and Lindquist, 2005; Pearl and Prodromou, 2006; Sharp and Workman, 2006; Workman et al., 2007). The biological functions of HSP90 have been identified. Its crystal structures indicated that HSP90 has four functional domains: a middle domain, an N-term domain, ATP/ADP-binding domain, and a C-term dimerization domain (Pearl and Prodromou, 2006). Based on the structural information of HSP90, a high-throughput screening was conducted which generated the active drug inhibitor: compound 7 (CCT018159) (Cheung et al., 2005; Smith et al., 2006; Sharp et al., 2007). The subsequently obtained co-crystal structure of HSP90-compound 7 (CCT018159) complex revealed that further modification of compound 7 (CCT018159) by replacing or adding certain functional groups could improve the pharmacokinetic properties. Moreover, replacing the methyl group to an amide group (VER-49009), changing pyrazolyl ring to isoxazole aromatic ring (VER-50589), and modifying some other chemical groups (see **Scheme 1C**) led to a potent effect in animal cancer models. Followed by toxicology and safety evaluation, Luminespib (NVP-AUY922) has been proved to be a strong HSP90 inhibitor which is now in clinical trials. More recently, Luminespib, a drug in phase one clinical trials, exhibited positive results for patients with ALK rearrangements (Felip et al., 2018). Luminespib (NVP-AUY922) also exhibited potent anti-tumor activity in lung adenocarcinomas targeting EGFR exon 20 insertion mutations and cellular models in a confirmatory clinical trial (Jorge et al., 2018; Piotrowska et al., 2018). Moreover, Luminespib (NVP-AUY922) serves as one of the components in anticancer combination therapies, which are now at different stages of clinical trials (Garcia-Carbonero et al., 2013; Rong and Yang, 2018). To depict how computational drug discovery facilitates to the development of anticancer drugs, we listed the FDA-approved anticancer drug in recent 3 years which was obtained from National Cancer Institute database (Heller, 1951) in **Table 3**.

**Table 3:** The list of FDA-approved anticancer drugs in recent 3 years from the National Cancer Institute database.

Name	Chemical Structure	Therapeutic area	Target and functiuon	Year of Approval
Alpelisib	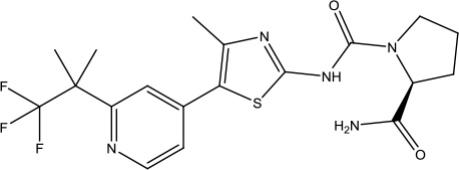	Breast cancer	PI3K inhibitor	2019 (Markham, 2019a)
Cladribine	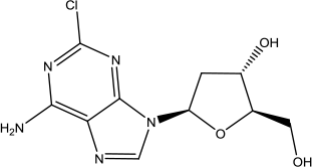	Hairy cell leukemia	Adenosine deaminase inhibitor	2019 (Bryson and Sorkin, 1993)
Darolutamide	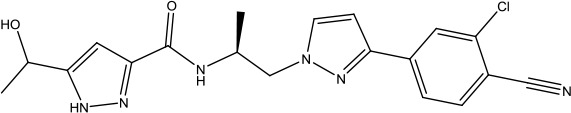	Prostate cancer	Androgen receptor inhibitor	2019 (Markham and Duggan, 2019)
Entrectinib	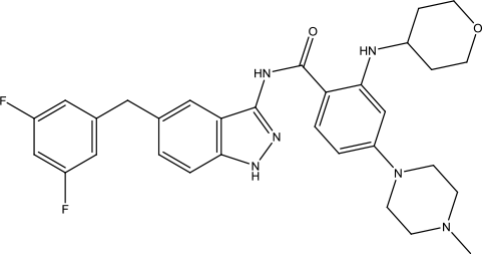	Non-small cell lung cancer and Solid tumors	Tyrosine kinase inhibitor	2019 (Al-Salama and Keam, 2019)
Erdafitinib	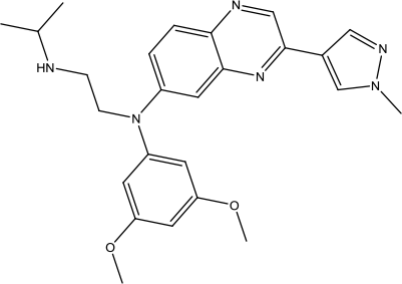	Urothelial carcinoma	FGFR tyrosine inhibitor	2019 (Markham, 2019b)
Fedratinib Hydrochloride	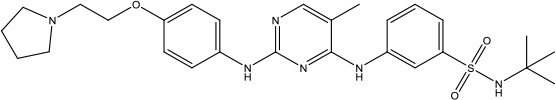	Myelofibrosis	Tyrosine kinase inhibitor	2019 (Zhang et al., 2014)
Selinexor	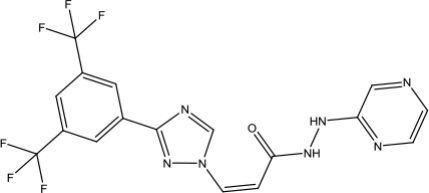	Multiple myeloma	Nuclear export inhibitor	2019 (Syed, 2019)
Zanubrutinib	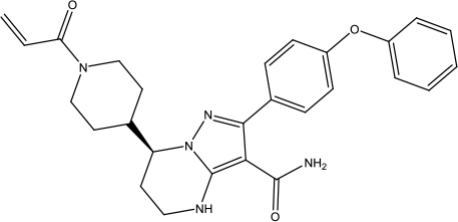	Mantle cell lymphoma	Bruton's tyrosine kinase inhibitor	2019 (Syed, 2020)
Abemaciclib	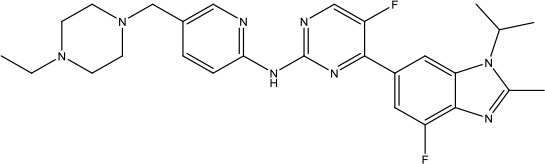	Breast cancer	Cyclin-dependent kinase inhibitor	2018 (Kim, 2017b)
Apalutamide	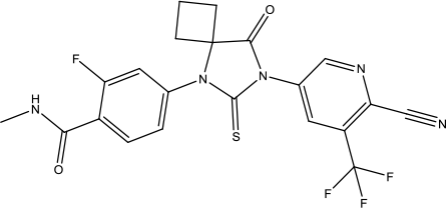	Prostate cancer	Androgen receptor inhibitor	2018 (Al-Salama, 2019)
Binimetinib	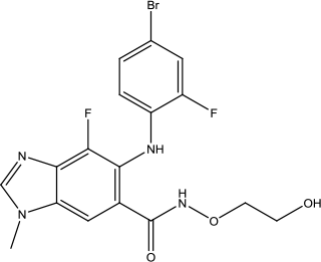	Melanoma	MEk1 and MEK2 inhibitor	2018 (Shirley, 2018)
Dacomitinib	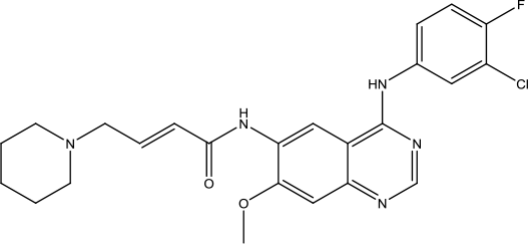	Non-small cell lung cancer	Oral kinase inhibitor	2018 (Sidaway, 2018)
Duvelisib	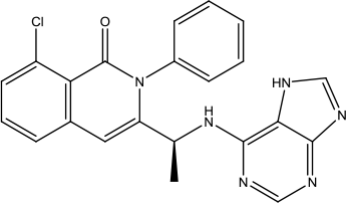	Chronic lymphocytic leukemia (CLL) and follicular lymphoma (FL)	PI3K Kinase inhibitor	2018 (Blair, 2018)
Encorafenib	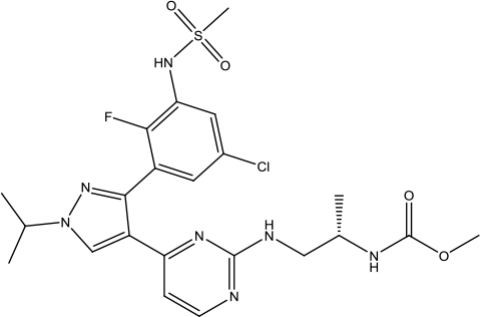	Colorectal cancer and Melanoma	BRAF Kinase inhibitor	2018 (Shirley, 2018)
Gilteritinib Fumarate	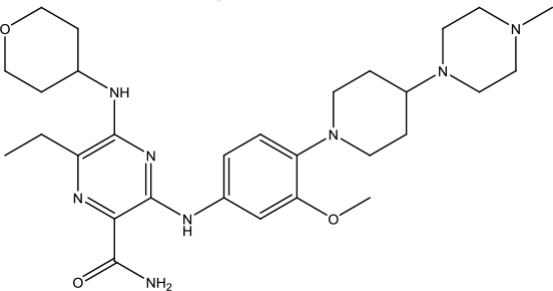	Acute myeloid leukemia	Tyrosine kinase inhibitor	2018 (Dhillon, 2019)
Glasdegib Maleate	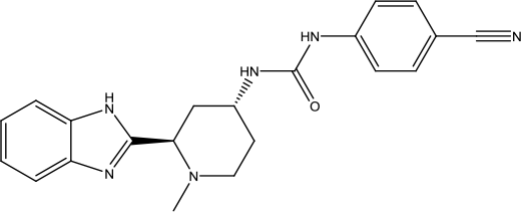	Acute myeloid leukemia	Hedgehog pathway inhibitor	2018 (Shaik et al., 2019)
Iobenguane I 131	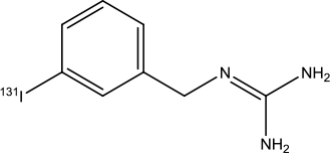	Pheochromocytoma	Radioactive therapeutic agent	2018 (Giammarile et al., 2008)
Ivosidenib	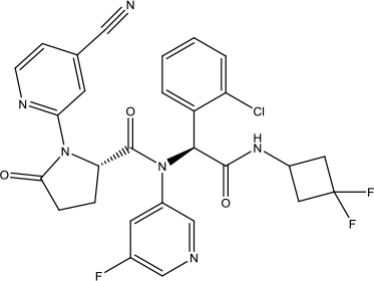	Acute myeloid leukemia	Isocitrate dehydrogenase-1 (IDH1) inhibitor	2018 (Dhillon, 2018)
Larotrectinib Sulfate	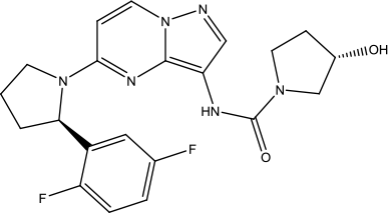	Solid tumors	Tropomyosin-related kinase (Trk) inhibitor	2018 (Gajdosik, 2017)
Lorlatinib	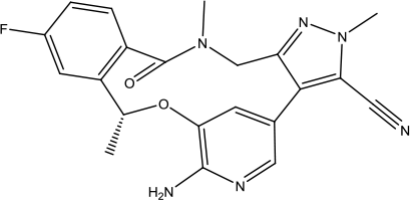	Non-small cell lung cancer	Tyrosine kinase inhibitor	2018 (Su et al., 2019)
Talazoparib Tosylate	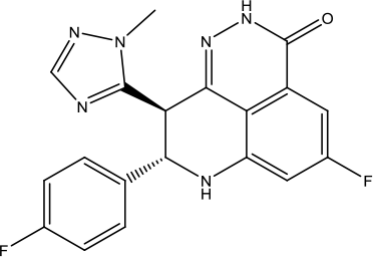	Breast cancer	Poly (ADP-ribose) polymerase (PARP) inhibitor	2018 (Eskiler, 2019)
Acalabrutinib	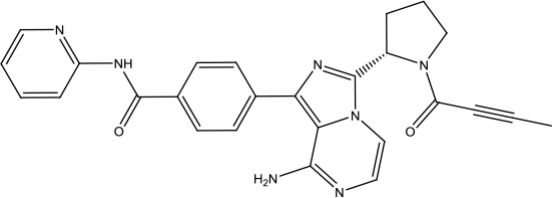	Chronic lymphocytic leukemia, small lymphocytic lymphoma, and mantle cell lymphoma	Bruton's tyrosine kinase inhibitor	2017 (Markham and Dhillon, 2018)
Brigatinib	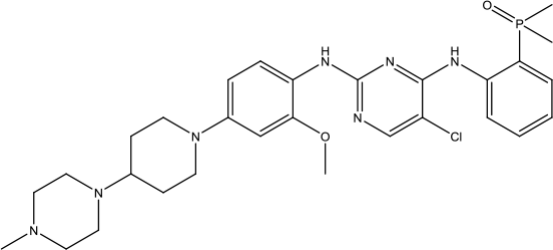	Non-small cell lung cancer	Anaplastic lymphoma kinase (ALK) and epidermal growth factor receptor (EGFR) kinase inhibitor	2017 (Markham, 2017a)
Copanlisib Hydrochloride	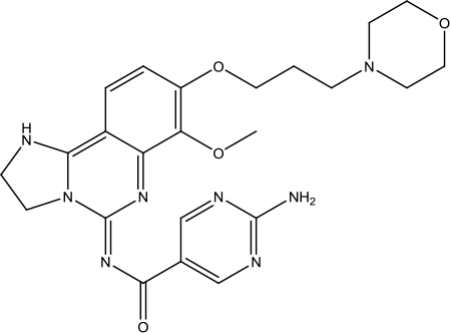	Follicular lymphoma	Phosphoinositide 3-kinase (PI3K) inhibitor	2017 (Markham, 2017b)
Enasidenib Mesylate	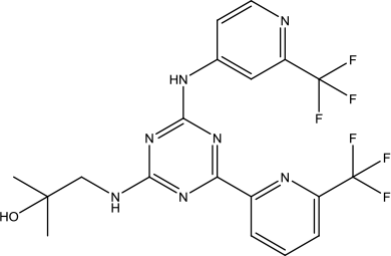	Acute myeloid leukemia	Isocitrate dehydrogenase-2 inhibitor	2017 (Gras, 2017)
Midostaurin	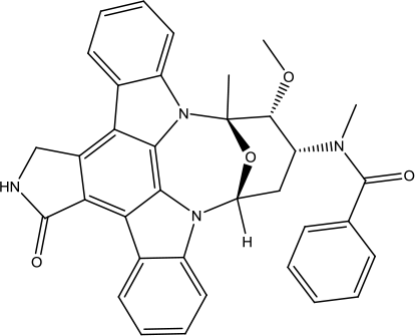	Acute myeloid leukemia	Synthetic indolocarbazole multikinase inhibitor	2017 (Kim, 2017a)
Neratinib Maleate	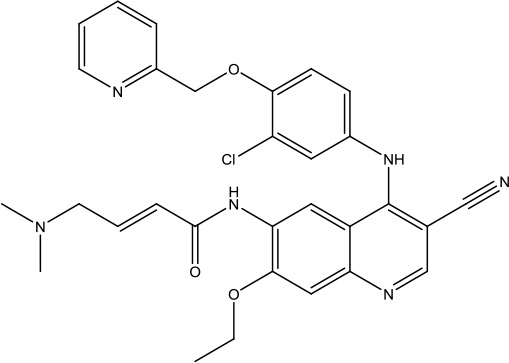	Breast cancer	Receptor tyrosine kinases (RTKs), Human epidermal growth factor receptor 2 (HER2; ERBB2), and Human epidermal growth factor receptor (EGFR) inhibitor	2017 (Kotecki et al., 2019)
Niraparib Tosylate Monohydrate	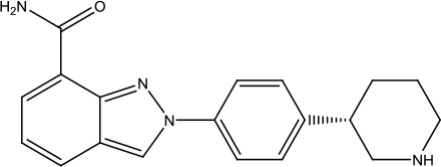	Recurrent epithelial ovarian, fallopian tube and primary peritoneal cancer	Poly (ADP-ribose) polymerase (PARP) inhibitor	2017 (Mittica et al., 2018)
Ribociclib	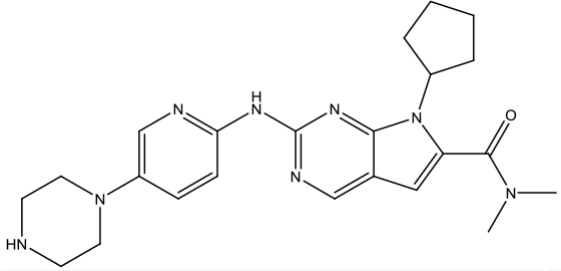	Breast cancer	Cyclin-dependent kinase (CDK) inhibitor	2017 (Syed, 2017)

We further manually screened the database to remove drugs that do not directly target cancer. Drugs for ameliorating conditions related to cancer or limiting side effects of cancer therapies are not listed in this short list. We then identified the FDA label of the drugs in the shortlist by searching in the U.S. National Library of Medicine database “DailyMed”. The FDA approval date, drug function, and therapeutic area are retrieved from DailyMed database.

## Conclusion and Perspective

Cancer has become a tangible threat to human health. About 9.6 million people are estimated to die from the various forms of cancer each year, according to a statistic report (Collaborators, 2019). Cancer has become the second-largest disease that causes human death (Reimann et al., 2020). However, developing a new drug molecule costs 12 years and 2.7 billion USD on average (Hauser et al., 2017). The drug development for cancer even becomes more complicated, especially considering the molecular pharmacology is still not well understood. Hence, the discovery and development of new drugs is considered very expensive and time-consuming. In this respect, computational methods could be constructive for performing different tasks including protein-interaction network analysis, drug-target prediction, binding site prediction, virtual screening, and many others. All these innovative methods could considerably facilitate the anti-cancer drug discovery. In recent years, with the advance of AI, more sophisticated methods, such as retro-synthetic routine plan, drug scaffold generation, drug binding affinity predictions, were developed. The useful predictions generated by computational models combined with experimental validations could further speed up the anti-cancer drug development.

## Author Contributions

SY designed the whole review. WC directed the completion of the review. AA, SW, QY, and YL were supportive during the review.

## Funding

This work was supported by the internal funding of Shenzhen Institutes of Advanced Technology, Chinese Academy of Sciences.

## Conflict of Interest

SY is the cofounder of AlphaMol Science Ltd.

The remaining authors declare that the research was conducted in the absence of any commercial or financial relationships that could be construed as a potential conflict of interest.
